# Gram-negative quorum sensing signalling enhances biofilm formation and virulence traits in gram-positive pathogen *Enterococcus faecalis*

**DOI:** 10.1080/20002297.2023.2208901

**Published:** 2023-05-11

**Authors:** Ana Parga, Daniel Manoil, Malin Brundin, Ana Otero, Georgios N. Belibasakis

**Affiliations:** a Department of Microbiology and Parasitology, CIBUS-Faculty of Biology, Universidade de Santiago de Compostela, Santiago de Compostela, Spain; b Division of Oral Diseases, Department of Dental Medicine, Karolinska Institutet, Huddinge, Sweden; c Division of cariology and endodontics, University Clinics of Dental Medicine, Faculty of Medicine, University of Geneva, Geneva, Switzerland; d Division of Endodontics, Department of Odontology, Umeå University, Umeå, Sweden

**Keywords:** *Enterococcus faecalis*, biofilm formation, endodontic infection, quorum sensing, Fsrc, cell-to-cell communication, acyl-homoserine lactones, gene expression, virulence, reverse-transcription quantitative PCR

## Abstract

Acyl-homoserine lactones (AHLs) are typical quorum-sensing molecules of gram-negative bacteria. Recent evidence suggests that AHLs may also affect gram-positives, although knowledge of these interactions remains scarce. Here, we assessed the effect of AHLs on biofilm formation and transcriptional regulations in the gram-positive *Enterococcus faecalis*. Five *E. faecalis* strains were investigated herein. Crystal violet was employed to quantify the biomass formed, and confocal microscopy in combination with SYTO9/PI allowed the visualisation of biofilms’ structure. The differential expression of 10 genes involved in quorum-sensing, biofilm formation and stress responses was evaluated using reverse-transcription-qPCR. The AHL exposure significantly increased biofilm production in strain ATCC 29212 and two isolates from infected dental roots, UmID4 and UmID5. In strains ATCC 29212 and UmID7, AHLs up-regulated the quorum-sensing genes (*fsrC*, *cylA*), the adhesins *ace*, *efaA* and *asa1*, together with the glycosyltransferase *epaQ*. In strain UmID7, AHL exposure additionally up-regulated two membrane-stress response genes (σ^V^, *groEL*) associated with increased stress-tolerance and virulence. Altogether, our results demonstrate that AHLs promote biofilm formation and up-regulate a transcriptional network involved in virulence and stress tolerance in several *E. faecalis* strains. These data provide yet-unreported insights into *E. faecalis* biofilm responses to AHLs, a family of molecules long-considered the monopole of gram-negative signalling.

## Introduction

*Enterococcus faecalis* is a facultative anaerobe, gram-positive bacterium that is commensal in the oral and gut microbiota of humans. Yet, outside of its niches, *E. faecalis* behaves as a major human opportunistic pathogen [1]. The bacterium is a leading cause of nosocomial infections, including urinary tract, surgical sites, endocardium and bloodstream infections [2,3]. Besides, in dental medicine, *E. faecalis* remains among the most frequently retrieved taxa from recurring dental root infections, thereby highlighting the remarkable ability of the species to survive antimicrobial procedures [4–7].

One crucial factor that allows *E. faecalis* to withstand bleak conditions and colonise multiple sites, is its capacity to rapidly adhere onto surfaces and form biofilms [8,9]. Biofilms confer a protective barrier against the penetration of antimicrobials and foster the development of persistor cells [10,11]. As a result, *E. faecalis* cells embedded within biofilms are estimated to be up to 1000-fold less susceptible to antimicrobials, which is clinically troublesome when considering that biofilms account for an estimated 65 to 80% of infections that require hospitalisation [12,13]. A better understanding of the factors underlying biofilm formation, and of ways to tackle these infections, remains therefore imperative.

Biofilm initiation and maturation are in part regulated by quorum sensing, i.e. a cell-density sensing mechanism regulated by the accumulation of signalling molecules in the vicinity of bacterial cells [14]. Binding of these molecules to their cognate receptors induces a series of signal transductions that coordinate gene expression in the microbial community. It is classically described that low-molecular-weight oligopeptides are involved in gram-positive signalling, as they easily diffuse through the peptidoglycan cell wall to bind to their cognate receptors anchored on the outer part of the cytoplasmic membrane. In contrast, gram-negatives rely on acyl-homoserine lactones (AHLs) to coordinate quorum-depending processes such as cell-to-cell communication and biofilm formation [14]. AHLs comprise a homoserine lactone (HSL) ring connected to an acyl chain of variable length, which renders the molecule hydrophobic and hence able to cross gram-negatives’ outer- and cytoplasmic membranes [14]. Nonetheless, recent evidence suggests that these intergeneric signalling boundaries may not be so distinct. Specifically, several reports show that interfering with AHLs signalling in polymicrobial biofilms may affect their taxonomic composition, including the relative abundance of gram-positive taxa [15–17]. These reports support an effect of AHLs (or AHL-like molecules) that extends to gram-positives, although the evidence remains indirect. Other reports on single gram-positive taxa, including *Listeria monocytogenes* and *Staphylococcus sciuri*, have observed a quantitative increase in their biomass upon exposure to AHLs [18,19]. In addition, exposure of planktonic *Staphylococcus aureus* to an oxo-substituted AHL (3-oxo-C_12_-HSL) was shown to decrease its expression of exotoxins, fibronectin adhesins and the agr virulence-associated regulon, while up-regulating an immunoglobulin-binding protein [20]. Whereas these reports further point towards an effect of AHLs beyond gram-negatives, the extent of this impact on gram-positive biofilms, and specifically whether AHLs may alter transcriptional regulations of biofilm formation and virulence-associated traits, remains scarcely addressed. Insights into these ecological interactions may help better understand how polymicrobial ecosystems, where gram-positives and -negatives thrive alongside, may impact virulence and survival strategies of pathogens such as *E. faecalis*.

Therefore, we aimed to examine the effects of different AHLs on the early biofilm formation and transcriptional regulations in *E. faecalis*. Specifically, biofilms of five different strains of *E. faecalis* exposed to either short- or long-chain AHLs were quantified using a crystal violet (CV) assay. Furthermore, *E. faecalis* biofilms were observed by confocal-laser scanning microscopy in combination with SYTO 9/propidium iodide (PI) staining. Finally, the differential expression of 10 virulence-associated genes involved in quorum sensing, biofilm formation and membrane-stress responses was assessed by reverse-transcription and quantitative PCR (RT-qPCR).

## Materials and methods

### Strains and culture conditions

Five *E. faecalis* strains were investigated here; collection strains ATCC 29212, the type strain ATCC 19433^T^, along with three isolates from infected dental root canals labelled UmID4, UmID5 and UmID7. Root canal isolates were obtained from Umeå bacterial collection (Division of Endodontics, Department of Odontology, Umeå University, SE). Clinical isolates were originally collected from both untreated and previously infected dental root canals ongoing endodontic treatment. In brief, root canal samples were plated onto bile/esculin/sodium azide agars dedicated to group D streptococcal isolation. Among the group D streptococci isolated (black colonies positive for esculin hydrolysis), *E. faecalis* cells were further sorted by testing for pyroglutamyl-aminopeptidase (PYR) and leucine-aminopeptidase (LAP) activities using methylumbelliferyl-associated substrates. PYR/LAP positive isolates that are taxonomically classified as *E. faecalis*, were additionally confirmed by species-specific quantitative PCR using the primers F: 5’-CCGAGTGCTTGCACTCAATTGG-3’ and R: 5’-CTCTTATGCCATGCGGCATAAAC-3’ that amplify a 138 bp amplicon on the 16S rRNA gene [21]. All *E. faecalis* strains and isolates were routinely cultured on Brain Heart Infusion (BHI) agar plates at 37°C.

### Preparation of acyl-homoserine lactones (AHLs)

The AHLs employed in this study were *N*-Butyryl-DL-homoserine lactone (C_4_-HSL; Fluka, Thermo Fisher Scientific, Waltham, US), *N*-(3-Oxobutyryl)-L-homoserine lactone (3-oxo-C_4_-HSL; University of Nottingham, Nottingham, GB), *N*-Hexanoyl-L-homoserine lactone (C_6_-HSL; Sigma-Aldrich, Merck KGaA, Darmstadt, DE), *N*-Oxohexanoyl-L-homoserine lactone (3-oxo-C_6_-HSL; University of Nottingham), *N*-Octanoyl-L-homoserine lactone (C_8_-HSL; Sigma-Aldrich), *N*-Decanoyl-L-homoserine lactone (C_10_-HSL; Sigma-Aldrich), *N*-Dodecanoyl-L-homoserine lactone (C_12_-HSL; Sigma-Aldrich), *N*-Tetradecanoyl-DL-homoserine lactone (C_14_-HSL; Fluka) and *N*-Octadecanoyl-L-homoserine lactone (C_18_-HSL; University of Nottingham).

AHLs were clustered in two groups according to their acyl chain length. The short-chain group included C_4_-HSL, OC_4_-HSL, C_6_-HSL, OC_6_-HSL and C_8_-HSL, whereas the long-chain group included C_10_-HSL, C_12_-HSL, C_14_-HSL and C_18_-HSL. Stock solutions were prepared for each AHL in acetonitrile. All AHLs were dissolved to a titre of 1 mg/mL and stored at −20°C, except C_18_-HSL that had to be dissolved to 0.1 mg/mL due to its lower solubility. Prior to each experiment, intermediate dilutions of these stock solutions were prepared in sterile water to generate mixes of the respective short- and long-chain AHLs, reaching a total concentration of 20 μM for each AHL.

### Generation of *E.faecalis* biofilms and quantification following AHL exposure

Bacteria retrieved from agars were inoculated into 8 mL BHI and incubated overnight at 37°C to generate suspension cultures. Overnight suspensions were pelleted by centrifugation (4’200 rpm, 5 min), the supernatant was removed and the pellet resuspended in fresh BHI to spectrophotometrically adjust bacterial density at OD_600 nm_ 0.5 (approx. 4 × 10^8^ CFU/mL) (UV-1800 spectrophotometer, Shimadzu, Kyoto, JP). Biofilms were generated onto the bottom of 24-well polystyrene plates (Sarstedt AG & Co. KG, Nümbrecht, DE). For that purpose, all wells were filled with 1.7 mL BHI broth, and 200 μL of the OD-adjusted *E. faecalis* suspension were then added to each well. One-hundred microliters of the short- or long-chain AHL mixes were finally poured into the corresponding wells, yielding a working concentration of 1 μM for each individual AHL in the mix (acetonitrile fractions between 0.1 and 0.5% v/v). Conditions therefore comprised *E. faecalis* biofilms exposed either to long- or short-chain AHLs. Control biofilms received sterile water with the same final fraction of acetonitrile. Plates were incubated during 6 h at 37°C.

Following biofilm incubation, culture media were removed by aspiration and biofilms were washed three times with a solution of phosphate buffer saline (PBS). All wells were then stained for 20 min in 2 mL of a 0.04% solution of crystal violet (CV) (Sigma-Aldrich). After staining, the excess dye was removed by aspiration and biofilms were again washed three times with PBS. The stained biofilms were homogenised in 2 mL of 33% acetic acid, and the CV incorporated in the biomass was measured at 595 nm.

### Confocal-laser scanning microscopy imaging

For confocal-laser scanning microscopy (CLSM) visualisation, *E. faecalis* biofilms were generated as described in the previous section, in the presence of either short- or long-chain AHL mixes. After 6 h of incubation, the biofilms were washed three times with PBS and immediately stained with two nucleic acid dyes; SYTO 9 and propidium iodide (PI) (LIVE/DEAD BacLight Bacterial Viability Kit, Thermo Fisher Scientific). The staining solution was prepared by mixing SYTO 9 (3.34 mM) and PI (20 mM) in a 1:1 proportion and further diluting it 150-fold in sterile PBS (pH 7.4), to final working concentrations of 11 μM for SYTO 9 and 66 μM for PI. Fifty microliters of the staining solution were poured onto each biofilm, and samples were incubated for 15 min at room temperature protected from light prior to image acquisition. Biofilms were observed using a Leica Stellaris 8 FALCON confocal microscope, equipped with the White Laser WLL2 and the objectives HC PL APO CS2 20×/0.75 Dry and the 40×/1.30 Oil (Leica Microsystems GmbH, Wetzlar, DE). SYTO 9 is membrane permeable and enters all bacterial cells, whereas PI only penetrates membrane-damaged cells. Yet, the SYTO 9 is displaced from the DNA upon PI entry due to its higher association constant with nucleic acids, thereby shifting the fluorescence of membrane-damaged cells from green to red [22]. Biofilms were observed using the laser excitation/emission bands ex. 499/em. 504–555 nm for SYTO 9 and ex. 561/em. 570–675 nm for PI.

Photomicrographs of the biofilms were acquired at 20× and 40× magnification. To calculate the area covered by biofilms, eight randomly selected fields (20×) in each condition were analysed with the Leica Application Suite X software (LAS X v.3.7.4, Leica Microsystems). To do so, the area irradiated by photons from both the SYTO 9 and PI channels was divided by the total field of view (FoV). The results express percentages of the area covered by *E. faecalis* biofilms in each condition.

### Assessment of AHLs effect on planktonic growth kinetics

To determine whether AHLs influence planktonic growth, *E. faecalis* proliferation was monitored in the presence of AHL mixes in 96-well plates (BRAND GMBH + Co. KG, Wertheim, DE). To do so, 170 μL of fresh BHI were inoculated with 20 μL of an *E. faecalis* suspension at OD_600 nm_ 0.5. Ten microliters of short- or long-chain AHL mixes were then added to reach a final working concentration of 1 μM for each AHL, as for biofilm experiments. Controls received sterile water with the same final fraction of acetonitrile. Incubation was performed under orbital shaking in a microplate reader (MultiSkan SkyHigh, Thermo Fisher Scientific) at 37°C. Optical density readings were performed at 600 nm every 10 min.

### RNA extraction and generation of a complementary DNA library

*E. faecalis* biofilms were grown and exposed to short- or long-chain AHLs as previously described herein. After 6 h of incubation, the biofilms were washed three times with pre-heated BHI (at 37°C) to remove loosely attached cells and immediately frozen at −80°C. RNA extractions were performed using the RiboPure Bacteria RNA Purification Kit (Thermo Fisher Scientific), following the manufacturer’s instructions. In brief, a solution of phenols and chaotropic salts was directly poured onto the frozen biofilms to already protect them from RNase activity while defrosting. Biofilms were detached by vigorously pipetting the solution in and out. The entire content of each well (detached biofilms in the phenol solution) was then transferred into zirconia beads-containing tubes, and bacteria were further lysed by bead-beating (TissueLyser II, Qiagen, Stockholm, SE). Chloroform was then added to separate the organic and aqueous phases. The aqueous phase was transferred to a clean tube to precipitate the RNA fraction with 99.9% molecular-grade ethanol. Further purification steps were performed using the provided glass-fibre columns, and RNA was finally eluted by passing twice 30 µL of the elution solution pre-heated at 65°C. DNase I treatment was ultimately performed to eliminate potential genomic DNA carryover. RNA yields were measured using a NanoDrop One/OneC Microvolume UV-Vis spectrophotometer (Thermo Fisher Scientific) and stored at −80°C.

Reverse-transcription (RT) was performed using the SuperScript IV VILO Master Mix (Thermo Fisher Scientific) according to the manufacturer’s instructions. Briefly, reactions were performed in 20 µL total volume that comprised 4 µL of the SuperScript IV VILO Master Mix, up to 16 μL of template RNA and completing up to the final volume with nuclease-free water when necessary (Thermo Fisher Scientific). The volume of input RNA templates was optimised to normalise the RNA quantity used in RTs for each strain. Reverse-transcription cycling comprised an annealing step at 25°C for 10 min, an RT step at 50°C for 10 min and a final enzyme inactivation step at 85°C for 5 min. No-RT controls were also run to ensure the absence of gDNA carryover. The generated cDNA was titrated fluorometrically by Qubit 3.2 (Thermo Fisher Scientific) using the dsDNA HS assay kit (Thermo Fisher Scientific) and stored at −20°C.

### Primer design

Primers were designed using the NCBI primer design tool by aligning sequences onto the reference genome of *E. faecalis* ATCC 29212 (GenBank accession number: GCA_000742975.1). Additional BLAST alignments against the reference genomes of strains ATCC 19433^T^ (GCA_000392875.1), V583 (GCA_000007785.1) and 39EA1 (GCA_003319815.1) were performed to bioinformatically verify that primers anneal to the target loci. Preliminary qPCRs were run to validate that each primer pair yielded a single amplicon product with a melting temperature (T_m_) corresponding to the predicted calculation. Table 1 displays the characteristics of each primer pair. Target genes were didactically clustered into three categories based on their function, i.e. quorum sensing-related genes, biofilm-related genes and membrane-stress responses.

**Table 1.** Primer pairs used in this study. The table includes the target genes, forward (F) and reverse (R) primer sequences, the length of their amplicon product, annealing temperature of the primer pairs as well as the gene annotations on the reference genome of *E. faecalis* ATCC 29212 (GenBank assembly accession: GCA_000742975.1), accessed on NCBI (28 September 2022).

Gene	Forward primer	Reverse primer	Amplicon size (bp)	T_m_ (°C) (F/R)	Gene annotation	Reference
*ace*	CGGATCGACAAGGAAGTGGT	CCTTGTTGCTCAAACTCGGC	109	59.75 60.04	DR75_173	[23]
*ebpA*	CGTTTCAGCCATTAGCCACG	CTTCACGCCAGGTGCTTTTC	74	59.90 60.04	DR75_165	This study
*efaA*	CCGTTACCAGAAGACATTGCG	TAAACCAGCCATTTCCGCCT	91	59.61 59.96	fimA	This study
*epaQ*	GCGATGTCTTTGGACACGAC	AATGAAGAGCGCCCGATAGC	81	59.56 60.60	DR75_1013	This study
*fsrC*	GCCAACAAACGAATCACAACC	ACCGCAAAGCAAGCAAAACT	98	58.88 59.82	DR75_811	[23]
σ^V^	AGCTTCTCGTTTCTTTTTACGCC	TGAAAAGGCACTGAAGGCCA	118	60.06 60.11	DR75_1896	[23]
*groEL*	GCACCGACTTTAACGACAGC	ACCAAATCGGCGAAACAACG	97	59.84 60.04	groL	This study
*asa1*	ACGCAATCCACAAAGTGCTG	CCTGCTTGCTGACTATCGCT	84	59.69 60.18	DR75_2177	This study
*cylM*	TGGTGACAGGGCTAGTACCAT	ACGCACTAAGGTTAACCCCTT	78	60.27 60.29	lanM *	This study
*cylA*	ACACGATTGCTCCAAGAGTGA	CCATCTGTCCCATCCATCACC	58	59.66 60.20	DR75_2957 *	This study
16S rRNA	CCGAGTGCTTGCACTCAATTGG	CTCTTATGCCATGCGGCATAA	137	62.58 60.61	DR75_111 (rRNA-16S)	[23]

* Sequence annotation on Plasmid 1 of *E. faecalis* ATCC 29212 (GenBank sequence accession: CP008815.1).

### qPCR workflow and relative quantification of target transcripts

Transcription levels of the targeted transcripts were assessed by qPCR. Reactions were performed in 20 µL total volume that comprised 10 µL of PowerUp SYBR Green Master Mix (Thermo Fisher Scientific), 6 µL of nuclease-free water, 1 µL of each forward and reverse primer (0.2 µM working concentration) and 2 µL of cDNA. qPCR constituents were mixed in MicroAmp Optical 96-Well Reaction Plates (Thermo Fisher Scientific), and reactions were run in an Applied BioSystems 7500 Fast Real-Time PCR thermocycler (Thermo Fisher Scientific). The amplification protocol consisted of two consecutive holding stages, at 50°C for 20 s and 95°C for 10 min, followed by 45 cycles of 95°C for 15 s and 60°C for 1 min. Melting curves were run at the end of each experiment with the following settings: 95°C for 15 s, 60°C for 1 min, a temperature transition slope of 1%, 95°C for 30 s and 60°C for 15 s.

The ∆∆Ct method was applied for relative quantification. The 16S rRNA gene was used as normaliser and the no AHLs controls as the calibrator conditions. The results are expressed as fold-change differences calculated as 2^−∆∆Ct^. Fold-changes in transcription lower than 0.5 or higher than 2 were considered to represent biologically relevant down- or up-regulations, respectively. The rationale for selecting a 2-fold threshold relied on methodological literature and on previous reports that adopted similar approaches [24,25]. All experiments were performed using four biological replicates.

### Statistical analysis

All statistical analyses were performed using GraphPad Prism 8 (GraphPad Software Inc., La Jolla, CA). The datasets’ fit to a normal distribution was tested by Kolmogorov–Smirnov or Shapiro–Wilk tests as appropriate. Accordingly, pairwise comparisons between CV assessments of short- or long-chain AHL treatments with the no AHL controls were performed using Mann–Whitney tests. Biofilm coverage areas calculated from CLSM photomicrographs were compared to the no AHL controls using t-tests. For the comparison of planktonic growth kinetics, nonlinear regression analyses were performed to obtain the best-fit values of each curve, which were then tested between conditions by one-way ANOVA and Dunnett’s multiple comparison tests. The 2^−∆∆Ct^ fold-change outputs between short- and long-chain AHLs were compared using Mann–Whitney tests. Significance was set at α = 0.05 for all analyses.

## Results

### Effect of AHL exposure on biofilm generation

**Figure 1.**
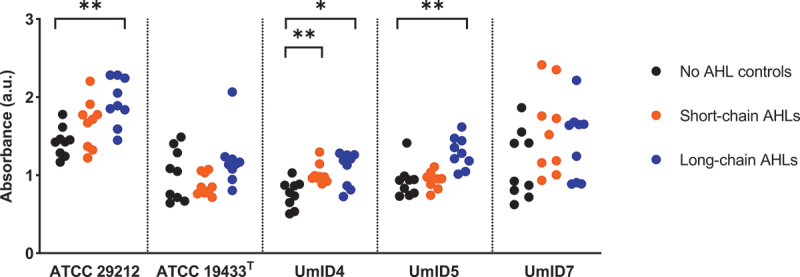
The effect of AHLs on *E. faecalis* biofilm formation. Scatter plots represent the amount of biofilm formed for each *E. faecalis* strain assessed by CV assays. The y-axis shows the amount of CV measured by spectrophotometric readings at 595 nm. Black dots show biofilms exposed to BHI only (no AHL controls), orange dots show biofilms exposed to short-chain AHLs and blue dots show biofilms exposed to long-chain AHLs. Each dot represents a replicate of three independent experiments performed in triplicate (*n* = 9). Statistical significance of pairwise comparisons between biofilms exposed to either short- or long-chain AHLs, and the no AHL controls are displayed as *p ≤ 0.05 and **p ≤ 0.01 (Mann–Whitney, α = 0.05).

AHLs were didactically clustered into either short- or long-chain AHLs based on their acyl chain length. *E. faecalis* biofilms were then generated in the presence of either short- or long-chain AHLs and stained with CV to explore potential quantitative effects on biofilm production. Figure 1 shows the CV measurements of these biofilms. The different strains of *E. faecalis* displayed varying abilities to produce biofilms, even in the absence of AHL exposure (negative controls). Upon AHL exposure, all strains exhibited a tendency to increase biomass production. Specifically, strain ATCC 29212 significantly increased its biofilm formation capacity by 36% upon exposure to long-chain AHLs (*p* = 0.0012). Significant increases of biomass were also observed in strain UmID4 following exposure to short- and long-chain AHLs, 33% (*p* = 0.0019) and 42% (*p* = 0.0153), respectively, as well as in UmID5 that exhibited a 40% increase after exposure to long-chain AHLs (*p* = 0.0012).

**Figure 2.**
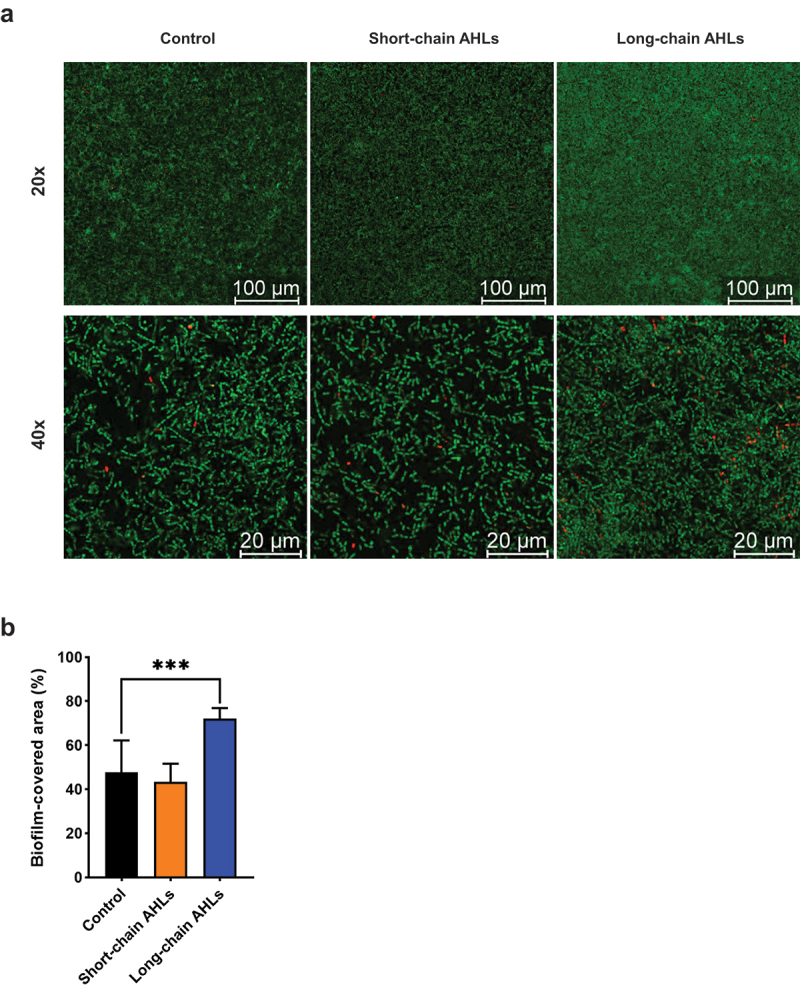
Confocal-laser scanning microscopy visualisation of *E. faecalis* biofilms. (a) Representative photomicrographs of *E. faecalis* ATCC 29212 biofilms grown without AHLs (left) and in the presence of either short- (middle) or long-chain AHLs (right). The upper photomicrographs display FoVs observed at 20× magnification and lower photomicrographs at 40× magnification. Scale bars represent 100 μm on 20× photomicrographs and 20 μm at 40× magnification. (b) Histograms represent the area covered by *E. faecalis* biofilms. Conditions are displayed on the x-axis. The y-axis expresses the percentage of biofilm-covered area relative to the total FoV (*n* = 8 fields at 20×). Statistical significance of pairwise comparisons between each AHL mix (short- or long-chains) and no AHL controls is displayed as ***p ≤ 0.001 (t-test, α = 0.05).

Figure 2 shows confocal microscopy observations of *E. faecalis* ATCC 29212 biofilms exposed to either short- or long-chain AHLs and stained with a combination of SYTO 9 and PI. Most cells appeared intact (SYTO 9-positive), and membrane-damaged cells (PI-positive) could be only sporadically observed independently of the presence of AHLs (Figure 2a). Measurements of biofilm-covered areas revealed that biofilms unexposed to AHLs extended over 48 ± 15% of the FoV, whereas biofilms exposed to long-chain AHLs displayed a significantly increased coverage to 72 ± 5% of the FoV (*p* = 0.0005) (Figure 2b).

### Growth kinetics of planktonic *E.faecalis* exposed to AHLs

**Figure 3.**
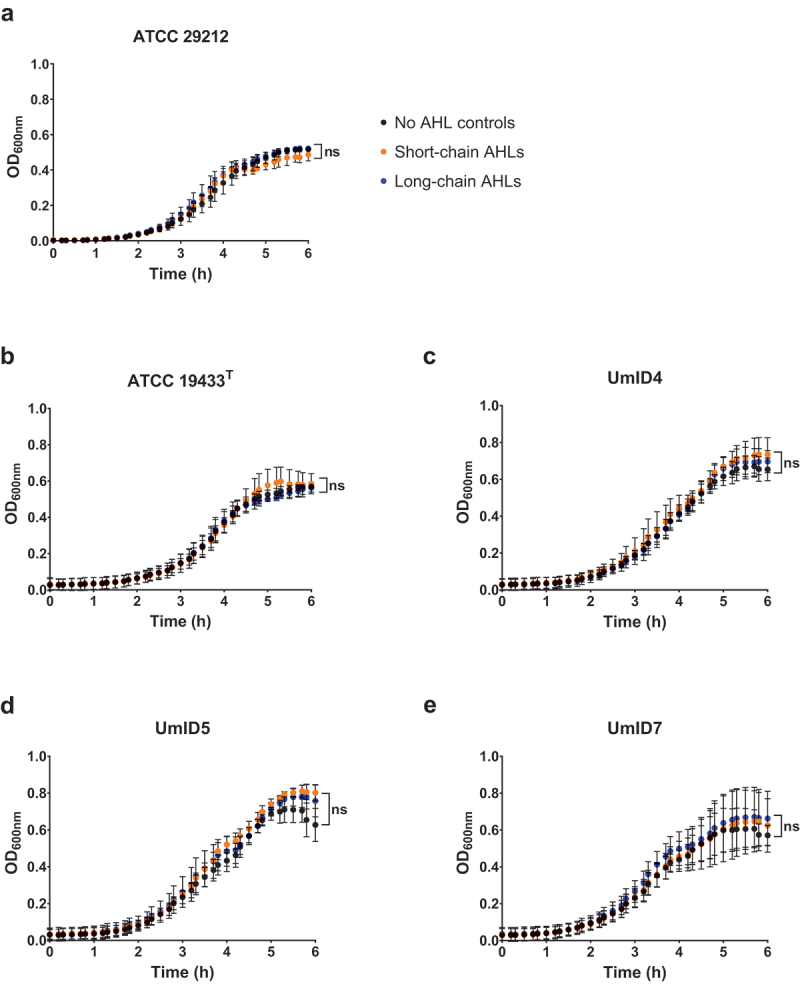
Planktonic growth curves of *E. faecalis* cultures exposed to AHLs. Graphs represent the growth of each *E. faecalis* strain monitored in time by optical density readings at 600 nm (y-axis). Black dots show controls unexposed to AHLs, whereas orange and blue dots show suspensions exposed to short- and long-chain, respectively. OD readings were acquired every 10 min. Each time-point measurement represents the mean of three independent experiments performed in triplicate (*n* = 9), and whiskers show SD. For each strain, the curves’ best-fit values were employed to compare the growth kinetics of suspensions exposed to either short- or long-chain AHLs with the no AHL controls (one-way ANOVAs, Dunnett’s multiple comparison tests, α = 0.05). ns: not significant.

To verify whether the increase in biomass observed resulted from a genuine effect of AHLs on biofilm formation and to rule out an indirect effect on cell proliferation, we monitored the kinetics of *E. faecalis* planktonic growth exposed to AHLs. Figure 3 shows the growth curves of planktonic suspensions of the five strains of *E. faecalis* in the presence of short- or long-chain AHLs (Figure 3a–e). Neither exposure to short- nor long-chain AHLs significantly altered planktonic growth in any of the strains.

### Differential gene expression in *E.faecalis* biofilms exposed to AHLs

**Figure 4.**
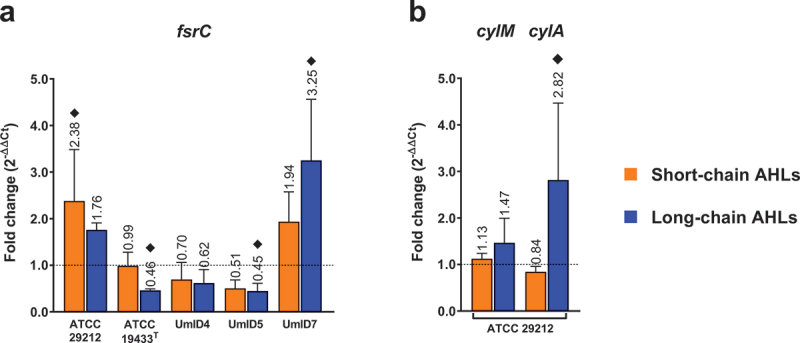
Differential expression of quorum sensing-related genes in biofilms of *E. faecalis* exposed to AHLs. Histograms display the transcription levels of *fsrC* (a) and *cylM* and *cylA* (b) as fold-changes calculated by the 2^−∆∆Ct^ algorithm (y-axis). The genes *cylM* and *cylA* were present only in strain ATCC 29212. Differential expression from biofilms exposed to short-chain ALHs is displayed by orange bars and that of biofilms exposed to long-chain ALHs by blue bars. Bars show mean fold-change values and whiskers represent SEM (*n* = 4 biological replicates). The value of each mean is indicated above whiskers. The dotted line at y = 1 defines the expression level of each gene in the calibrator condition, normalised to the expression of the 16S rRNA gene. Black diamonds (♦) mark fold-changes in transcripts levels that were considered biologically relevant. Statistical comparisons between conditions were assessed with Mann–Whitney tests (α = 0.05).

**Figure 5.**
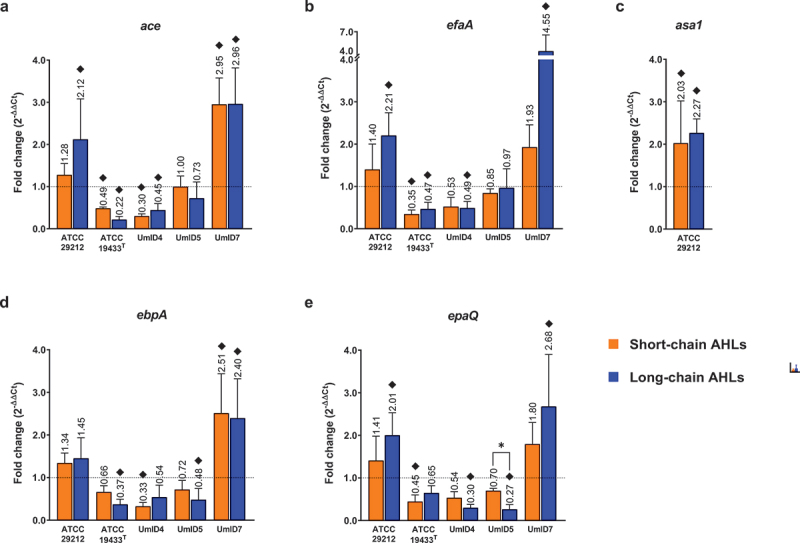
Differential expression of biofilm-related genes in biofilms of *E. faecalis* exposed to AHLs. Histograms display the transcription levels of *ace* (a), *efaA* (b), *asa1* (c), *ebpA* (d) and *epaQ* (e) as fold-changes calculated by the 2^−∆∆Ct^ algorithm (y-axis). Expression levels of *efaA* are shown on a split y-axis that covers fold-changes from 0 to 4 on its lower segment and from 4 to 7 on its upper segment. The gene *asa1* was present only in strain ATCC 29212. Differential expression from biofilms exposed to short-chain ALHs is displayed by orange bars and that of biofilms exposed to long-chain ALHs by blue bars. Bars show mean fold-change values and whiskers represent SEM (*n* = 4 biological replicates). The value of each mean is indicated above whiskers. The dotted line at y = 1 defines the expression level of each gene in the calibrator condition, normalised to the expression of the 16S rRNA gene. Black diamonds (♦) mark fold-changes in transcripts levels that were considered biologically relevant. Statistical comparisons between conditions are displayed as *p ≤ 0.05 (Mann–Whitney tests, α = 0.05).

**Figure 6.**
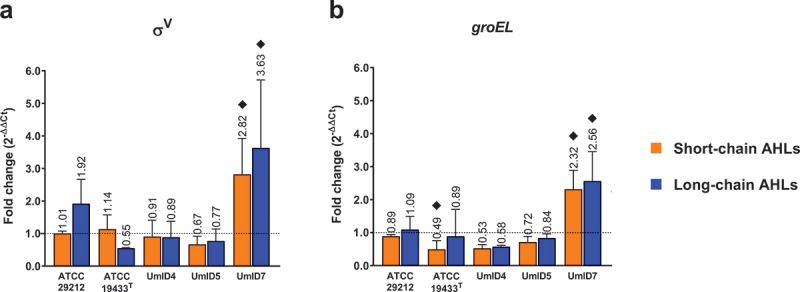
Differential expression of membrane-stress-related genes in biofilms of *E. faecalis* exposed to AHLs. Histograms display the transcription levels of σ^V^ (a), and *groEL* (b) as fold-changes calculated by the 2^−∆∆Ct^ algorithm (y-axis). Differential expression from biofilms exposed to short-chain ALHs is displayed by orange bars and that of biofilms exposed to long-chain ALHs by blue bars. Bars show mean fold-change values and whiskers represent SEM (*n* = 4 biological replicates). The value of each mean is indicated above whiskers. The dotted line at y = 1 defines the expression level of each gene in the calibrator condition, normalised to the expression of the 16S rRNA gene. Black diamonds (♦) mark fold-changes in transcripts levels that were considered biologically relevant. Statistical comparisons between conditions were assessed with Mann–Whitney tests (α = 0.05).

For gene expression experiments, the transcripts assessed were grouped into three categories based on their functions. Quorum sensing-related genes are shown in Figure 4, biofilm-associated genes are shown in Figure 5 and genes associated with membrane-stress responses in Figure 6. Down-regulations <0.5-fold and up-regulations >2-fold were considered biologically relevant differences in transcription levels.

Three transcripts representative of the two quorum sensing systems best described in *E. faecalis* were assessed, operons fsr and cyl. The main operon, fsr, encodes a two-component system that senses cell density and regulates virulence. The transmembrane histidine kinase, *fsrC*, senses the presence of gelatinase-biosynthesis activating pheromone (GBAP) and activates the transcription factor, *fsrA*. Downstream genes include *fsrC* itself, and transcripts encoding the membrane protein FsrB and the pro-peptide FsrD. FsrD is the precursor of GBAP, generated after processing by FsrB [26]. Herein, *fsrC* was up-regulated by 2.4-fold in strain ATCC 29212 after exposure to short-chain AHLs. Similarly, *fsrC* displayed a 3.3-fold up-regulation in strain UmID7 after exposure to long-chain AHLs (Figure 4a). On the other hand, the cytolysin operon regulates the production of a lantibiotic that results from the pore-forming activity of CylL_L_ and CylL_S_. These peptides require post-translation modifications by CylM and CylA to be active [26]. Herein, the cyl operon was only present in strain ATCC 29212. Among the two genes assessed, *cylM* and *cylA*, the latter displayed a 2.8-fold up-regulation after exposure to long-chain AHLs (Figure 4b).

Among biofilm-related genes, the collagen adhesin-encoding *ace* appeared 2.1-fold up-regulated in strain ATCC 29212 after exposure to long-chain AHLs. More important up-regulations were observed in strain UmID7, after exposure to short- (2.95-fold) and long-chain AHLs (3-fold) (Figure 5a). Contrastingly, the *ace* transcript also displayed down-regulations in strains ATCC 19433^T^ and UmID4 following AHL exposure. The endocarditis antigen A (*efaA*) showed relevant up-regulations upon exposure to long-chain AHLs, reaching 2.2-fold in strain ATCC 29212 and 4.6-fold in strain UmID7 (Figure 5b). The aggregation substance transcript *asa1* was only present in strain ATCC 29212, in which it was moderately up-regulated upon exposure to both short- (2-fold) and long-chain AHLs (2.3-fold) (Figure 5c). The pili-encoding transcript *ebpA* showed relevant up-regulations in strain UmID7 in response to short- (2.5-fold) and long-chain AHLs (2.4-fold), whereas down-regulations were observed in strains ATCC 19433^T^, UmID4 and UmID5 (Figure 5d). Finally, the hypothetical glycosyltransferase *epaQ* exhibited 2- and 2.7-fold up-regulation in strains ATCC 29212 and UmID7, respectively, in response to long-chain AHLs. Inversely, exposure to long-chain AHLs in UmID4 and UmID5 rather down-regulated *epaQ* expression to 0.3-fold in both strains (Figure 5e).

Among genes related to membrane-stress responses, the extra-cytoplasmic sigma-factor V (ECF-σ^V^) appeared relevantly up-regulated in strain UmID7 only, upon exposure to both short- (2.8-fold) and long-chain AHLs (3.6-fold) (Figure 6a). Similarly, the chaperone *groEL* was up-regulated in strain UmID7 after exposure to both short- (2.3-fold) and long-chain AHLs (2.6-fold) (Figure 6b).

## Discussion

The current study investigated the impact of AHL exposure on biofilm growth and on the differential expression of virulence-related genes in five strains of *E. faecalis*. Our data demonstrate that exposure to long-chain AHLs more specifically, promoted biofilm formation in three of the strains. Besides, exposure to short- and long-chain AHLs induced the up-regulation of a network of virulence- and biofilm-associated genes in two strains. To our knowledge, this report is the first to demonstrate biomass modifications and differential gene expression within *E. faecalis* biofilms in response to AHLs. These findings hold clinical and ecological relevance since *E. faecalis* naturally thrives in polymicrobial biofilms alongside gram-negative taxa [27–29]. Furthermore, these results may contribute to explain the more virulent phenotypes observed in polymicrobial communities, often concealed in single-species cultures [30].

In this study, AHLs were clustered into either short- or long-chains based on their acyl residue length. The reason for clustering AHLs into short and long molecules relied on an ecological rational. Evidence shows indeed that typical AHL receptors of the LuxR-type may be promiscuously activated by AHLs of similar length, so that several structurally related AHLs of the same cluster likely drive similar biological functions [31–33]. These considerations are typically exemplified in the gram-negative *Chromobacterium subtsugae*, in which several short-chain AHLs (C_4_ – C_8_) induce violacein production, whereas long-chain AHLs (C_10_ – C_16_) act as competitive antagonists [31]. This dichotomy between short- and long-chain AHLs is also present in other gram-negatives [34] and more intriguingly has also been observed in the gram-positive *S. aureus* that appeared to only react to long-chain AHLs (oxo-C_12_) [20]. Interestingly, our results further support such dichotomy in *E. faecalis* since mostly long-chain AHLs (C_10_ – C_18_) were shown to enhance biofilm formation and differentially regulate gene expression. Yet, because no gram-negative homologous AHL receptors are described in gram-positives, the mechanisms accounting for such AHL specificity in *E. faecalis* remain elusive.

This study assessed the impact of AHLs on *E. faecalis* early biofilm formation (6 h) to identify potential effects on adhesion and microcolony initial formation steps. It has indeed been shown that the role of quorum sensing in biofilm formation intervenes as early as 3 h in *E. faecalis* [35]. Furthermore, exposing *E. faecalis* cells to AHLs throughout the entire biofilm formation process circumvented penetrability issues within the biofilm extracellular matrix and ensured that all bacterial cells were equally exposed to AHLs. Control biofilms unexposed to AHLs displayed varying biomass amounts, which is in line with previous reports demonstrating differences in biofilm production among various *E. faecalis* strains [36,37]. Despite different biofilm-generation profiles, the addition of long-chain AHLs similarly affected three of the strains, i.e. the ATCC 29212, UmID4 and UmID5, which increased their biomass by 33 to 42%. This biofilm-promoting effect of long-chain AHLs was further corroborated herein by confocal observations of strain ATCC 29212, which displayed increased biofilm coverage by ~22%. Such degree of biomass increase fairly aligns with previous reports that also investigated the effect of AHLs on the biofilm formation abilities of two gram-positives, namely *Staphylococcus sciuri* and *Listeria monocytogenes*, and that reported biomass increases in the range 24–47% [18,19]. Remarkably, the biomass increases observed herein appear triggered by a direct effect of AHLs on biofilm production, rather than an indirect effect on cell proliferation, since the planktonic cultures of *E. faecalis* were unaltered by AHLs’ addition.

To gain deeper insights into the effects of AHLs on *E. faecalis* biofilms, current investigations evaluated the differential expression of 10 key transcripts involved in quorum sensing, biofilm formation and membrane stress responses. Overall, one observed important heterogeneity in transcriptional regulations between strains, which is in line with previous reports showing high phenotypical variations among *E. faecalis* strains [23,37]. Enterococci are indeed notable for their large genome plasticity owing to the presence of several mobile elements, genes present in different copy numbers and differentially regulated operons, which together may underlie the heterogeneity observed [38,39]. Despite these inter-strain variations, several noteworthy regulations in gene expression were observed.

Among the quorum sensing-related genes investigated, our data show an up-regulation of the cyl operon in strain ATCC 29212, suggesting an induction of lytic activity. The ability to form pores into eukaryotic cells, as well as competing bacterial counterparts, is one main virulence trait of this species [26,40]. More importantly, the histidine kinase membrane sensor *fsrC* also displayed relevant up-regulations in strains ATCC 29212 and UmID7, indicating an up-regulation of the fsr operon. One main role of the fsr operon is the expression of *gelE*. GelE is a zinc-metalloprotease able to hydrolyse haemoglobin, collagen, fibrine, C3a and C5a and is hence pivotal to *E. faecalis* virulence [35,41,42]. Whereas the mechanism underlying the observed activation of fsr upon AHL exposure cannot be inferred from our data, it may be relevant to mention that GBAP, the natural autoinducer of the fsr operon, also exhibits a lactone ring structurally similar to that of AHLs [43]. A potential cross-reactivity between lactone-containing peptides and AHLs could therefore be suspected to underlie current observations [44,45]. Concomitantly to these fsr up-regulations, our observations also showed significant increases in biomass in strain ATCC 29212 and a similar trend in strain UmID7, thereby linking the fsr up-regulation in these strains with increased biofilm formation upon AHL exposure. These observations are in agreement with previous reports that showed fsr to also regulate the transcription of genes outside the operon. These fsr-regulated genes mediate the excretion of exopolysaccharides and the expression of several adhesins involved in biofilm formation [46,47].

In this line, it is noteworthy that strains ATCC 29212 and UmID7 also exhibited up-regulations of several adhesins (*ace*, *efaA*, *ebpA* and *asa1*), as well as of the glycosyltransferase *epaQ*, upon AHL exposure. These adhesins mediate the adhesion of *E. faecalis* cells onto both abiotic surfaces, such as catheters, and biological substrates, such as collagen, endothelial cells or fibrin, and thereby highly contribute to pathogenicity during host invasion [48–51]. Additionally, the glycosyltransferase *epaQ* up-regulated herein was shown to trigger the production of cell wall-associated rhamnopolysaccharides that endow *E. faecalis* biofilms with antibiotic resistant properties [52]. Altogether, these data strongly suggest that AHL exposure may induce the expression of several adhesins associated with increased virulence and the formation of more recalcitrant biofilms. The presence of slight down-regulations in the other strains assessed must, however, also be acknowledged, indicating substantial inter-strain variations among these transcriptional regulations.

This study also measured the differential expression of two membrane stress-responses genes shown to enhance stress tolerance and virulence; σ^V^ and *groEL*. Current results showed up-regulations of these transcripts in strain UmID7 only. The σ^V^ subunit functions similarly to a two-component system, in which its cognate anti-σ-factor RsiV senses environmental stresses, typically lysozyme, and releases σ^V^ in the cytoplasm [53]. The released σ^V^ integrates the RNA polymerase holoenzyme and directs the transcription of specific sets of genes involved in stress coping. The second transcript, *groEL*, is a molecular chaperone involved in the stabilisation and re-folding of membrane proteins following stress [54]. GroEL was shown to mediate survival to heat, to osmotic and photo-oxidative stresses as well as to fluoroquinolones exposure [54–56]. The up-regulations of these transcripts in the strain UmID7 may have been triggered by a membrane instability caused by the incorporation of AHLs within the cytoplasmic membrane. There is indeed evidence demonstrating that the hydrophobicity of AHLs allows them to partition within the phospholipidic bilayers of gram-positive bacteria [20].

Whereas this study identified novel transcriptional co-regulations between quorum sensing operons and virulence-related genes upon AHL exposure, it also has limitations. When investigating the modulating effects of AHLs on *E. faecalis* biofilms, the use of AHL-mixes, as opposed to single AHL molecules, may have precluded the identification of single AHL effectors. Yet, such approach was selected in an effort to simulate complex polymicrobial environments in which *E. faecalis* normally thrives and therefore to more closely approximate naturally ensuing regulations. In this line, current qPCR investigations were designed to target a pre-selected set of key transcripts, although this approach may not uncover the full spectrum of underlying regulations, unlike an -omics approach may have.

Within the limitations of this study, our data indicate that exposure to AHLs, long-chains especially, promotes biofilm formation in several *E. faecalis* strains. More importantly, current results show that AHL exposure induced the expression of a network of genes associated with increased virulence, enhanced host invasion properties and biofilm generation, as well as increased stress tolerance. Whereas these data acknowledge important inter-strain variations, they provide yet-unreported insights into *E. faecalis* biofilm responses to AHLs, a family of signalling molecules long thought to be the monopole of gram-negative’s communication. Altogether, these data may contribute to understand how gram-positive taxa have evolved to respond to intergeneric cues in polymicrobial biofilms.
